# Complete chloroplast genome sequence of a Dutch cultivar of *Chrysanthemum*, *Chrysanthemum morifolium* ‘Orizaba’ (Asteraceae)

**DOI:** 10.1080/23802359.2021.1936672

**Published:** 2021-06-14

**Authors:** Hanhan Xia, Yufen Xu, Jiahao Zhang, Zhenqi Huang, Honghui Luo, Zhaoyuan Ye, Hougao Zhou

**Affiliations:** College of Horticulture and Landscape Architecture, Zhongkai University of Agriculture and Engineering, Guangzhou, China

**Keywords:** *Chrysanthemum*, chloroplast genomes, phylogenetic analysis, Orizaba

## Abstract

The complete plastid genome of *Chrysanthemum morifolium* ‘Orizaba’, a cultivar from Holland, was determined and analyzed in this work. It is a circular chromosome and has a length of 151,060 bp. The LSC and SSC of 82,858 bp and 18,294 bp were separated by two IRs of 24,954 bp. The chloroplast genome of *C. morifolium* ‘Orizaba’ contains 125 genes, including 83 protein-coding genes, eight ribosomal RNA genes, and 34 transfer RNA genes. Phylogenetic analysis showed that *C. morifolium* ‘Orizaba’ clustered together with other *Chrysanthemum* varieties in the family Asteraceae. The plastome is useful for the elucidation of phylogenetics and evolution in the Asteraceae and *Chrysanthemum* varieties.

*Chrysanthemum* (*C. morifolium*), classified in the Asteraceae, is one of the most popular and economically important floricultural crops in the world. It is noted for its ornamental value, edibility, tea, and medicinal uses, and has approximately 1600 years of cultivation history (Dowrick and El-Bayoumi 1966; Zhang et al. 2010). Cultivated chrysanthemums are highly heterozygous, with complex genetic backgrounds coming from multiple crosses over hundreds of years (Su et al. 2019). Understanding the plant chloroplast genome of the different *Chrysanthemum* cultivars is essential for the identification of commercial cultivars and the determination of their purity. In this study, we report and characterize the chloroplast genomes of a cultivar from Holland, *Chrysanthemum morifolium* ‘Orizaba’, which is a popular spray cut flower in the market.

Fresh leaves of *C. morifolium* ‘Orizaba’ were collected from the mature plant in the green house of Zhongkai University of agriculture and engineering (22°40′18.69″N, 113°15′5.37″E Guangzhou, China) and the specimen and the DNA are stored in Zhongkai University of Agriculture and Engineering (http://www.zhku.edu.cn/; xiahanhan@zhku.edu.cn under the voucher number ALSB-31-1_BDSW200007023-1a). After DNA extraction, a library with the insertion size of 350 bp was constructed, and high-throughput DNA sequencing (paired-end 150 bp) was performed on an Illumina X10 platform (San Diego, CA). In total, 10 of G raw reads were obtained for de novo assembly of the cp genome using NOVOPlasty (Dierckxsens et al. 2017), and then annotated in Plann (Huang and Cronk 2015) and GeSeq (https://chlorobox.mpimp-golm.mpg.de/geseq.html) using the default parameters. The complete genome sequence *C. morifolium* ‘Orizaba’ was submitted to NCBI GenBank under the accession number MT976166.

The complete cp genome of *C. morifolium* ‘Orizaba’ is 151,060 bp in length and contains, two short inverted repeat (IRa and IRb) regions of 24,954 bp, a large single-copy (LSC) region of 82,858 bp, and a small single-copy (SSC) region of 18,294 bp. The overall GC content of cp genome is 37.45%. The cp genome of *C. morifolium* ‘Orizaba’ contained 125 genes, including 83 protein-coding, 34 rRNA, and eight tRNA genes. Most of these genes do not contain introns; however, 16 genes contained one intron, and two genes (*clpP* and *ycf3*) contained two introns. All genes occurred as a single copy, except the 16 duplicated genes in the IR regions.

A phylogenetic analysis was carried out using cp genome sequences of *C. morifolium* ‘Orizaba’ and other 17 species/varieties (including duplicates) within the Asteraceae and nine representatives from the Rubiaceae, Caprifoliaceae, and Campanulaceae, which are related to the Asteraceae (Figure 1). The sequences were aligned using MAFFT 7 (Katoh and Standley 2013), and a maximum-likelihood tree was constructed using RAxML 8.2.12 (Stamatakis 2014) with 1000 bootstrap replicates and the nucleotide substitution model GTR + GAMMA. Phylogenetic analysis revealed that *C. morifolium* ‘Orizaba’ was grouped with other *Chrysanthemum* varieties (Figure 1), which indicate these varieties share a close related maternal donor or most recent ancestor. This result is in agreement with previous studies (Tyagi et al. 2019; Xia et al. 2021). In conclusion, this complete cp genome will enrich the genetic resource for future classification studies on *C. morifolium* and species in the Asteraceae.

**Figure 1.**
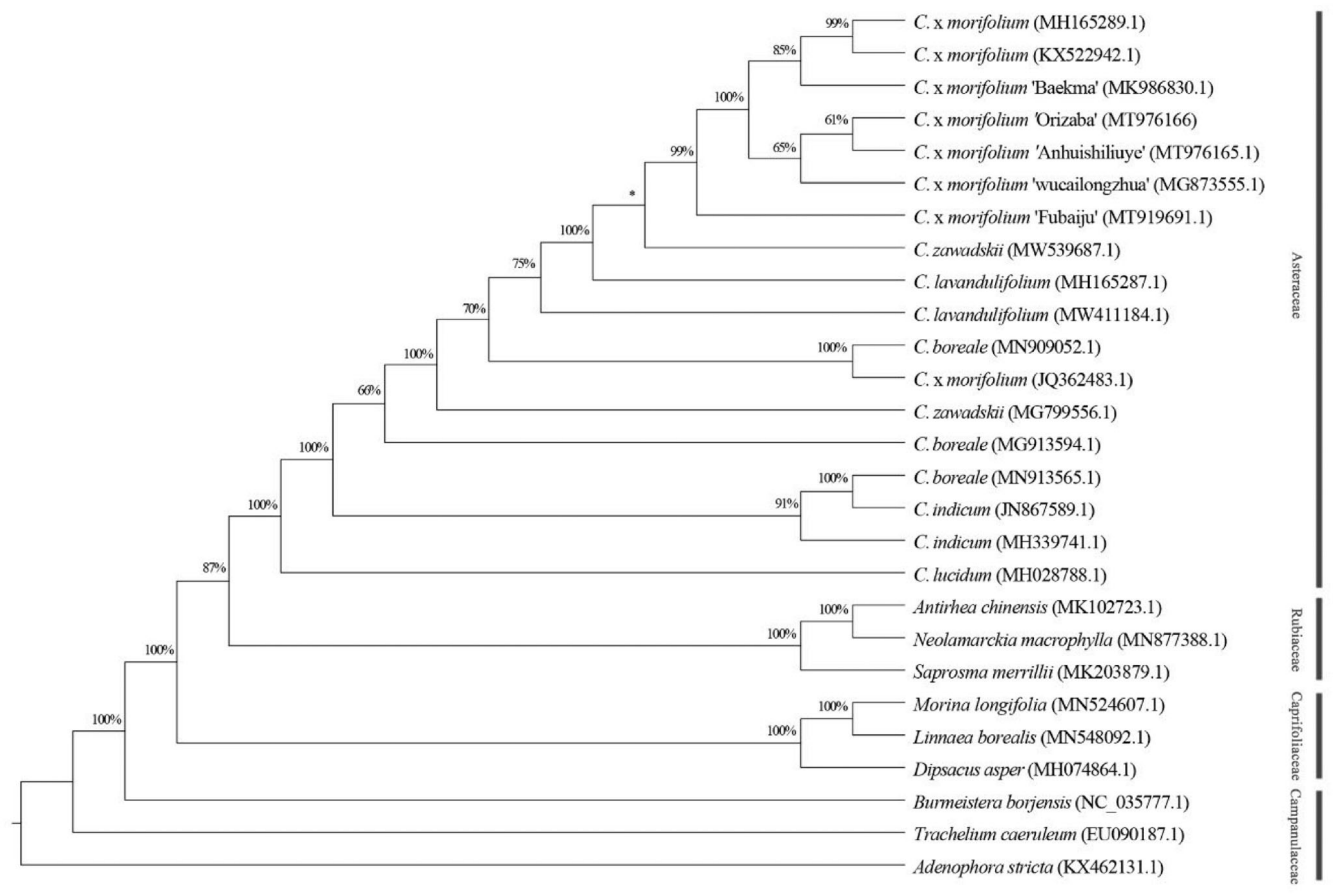
Phylogeny of *Chrysanthemum morifolium* ‘Orizaba’ and, 17 species/varieties (including duplicates) within family Asteraceae, and representatives from the Rubiaceae, Caprifoliaceae, and Campanulaceae. Maximum-likelihood tree was constructed with complete chloroplast genome sequences and 1000 bootstrap replicates. The scale bar represents the number of substitutions per site. *Bootstrap lower than 50%.

## Data Availability

The data that support the findings of this study are available in NCBI at https://www.ncbi.nlm.nih.gov/, the reference number is MT976166. The associated BioProject, SRA, and Bio-Sample numbers are PRJNA682858, SRR14506602, and SAMN19113560, respectively.
